# From two stages to one: acceleration of the induced membrane (Masquelet) technique using human acellular dermis for the treatment of non-infectious large bone defects

**DOI:** 10.1007/s00068-019-01296-x

**Published:** 2020-01-13

**Authors:** René Danilo Verboket, Maximilian Leiblein, Maren Janko, Alexander Schaible, Jan Claas Brune, Katrin Schröder, Myriam Heilani, Charlotte Fremdling, Yannic Busche, Tanja Irrle, Ingo Marzi, Christoph Nau, Dirk Henrich

**Affiliations:** 1 Department of Trauma-, Hand and Reconstructive Surgery, University Hospital Frankfurt, Frankfurt, Germany; 2 German Institute for Cell and Tissue Replacement (DIZG gemeinnützige GmbH), Berlin, Germany; 3 Center of Physiology, Cardiovascular Physiology, University Hospital Frankfurt, Frankfurt, Germany

**Keywords:** Induced membrane technique, Masquelet technique, Acellular dermis

## Abstract

**Introduction:**

The induced membrane technique for the treatment of large bone defects is a two-step procedure. In the first operation, a foreign body membrane is induced around a spacer, then, in the second step, several weeks or months later, the spacer is removed and the Membrane pocket is filled with autologous bone material. Induction of a functional biological membrane might be avoided by initially using a biological membrane. In this study, the effect of a human acellular dermis (hADM, Epiflex, DIZG gGmbH) was evaluated for the treatment of a large (5 mm), plate-stabilised femoral bone defect.

**Material and Methods:**

In an established rat model, hADM was compared to the two-stage induced membrane technique and a bone defect without membrane cover. Syngeneous spongiosa from donor animals was used for defect filling in all groups. The group size in each case was *n* = 5, the induction time of the membrane was 3–4 weeks and the healing time after filling of the defect was 8 weeks.

**Results:**

The ultimate loads were increased to levels comparable with native bone in both membrane groups (hADM: 63.2% ± 29.6% of the reference bone, *p* < 0.05 vs. no membrane, induced membrane: 52.1% ± 25.8% of the reference bone, *p* < 0.05 vs. no membrane) and were significantly higher than the control group without membrane (21.5%). The membrane groups were radiologically and histologically almost completely bridged by new bone formation, in contrast to the control Group where no closed osseous bridging could be observed.

**Conclusion:**

The use of the human acellular dermis leads to equivalent healing results in comparison to the two-stage induced membrane technique. This could lead to a shortened therapy duration of large bone defects.

## Introduction

The surgical treatment of long-range bone defects caused by traumas with high force or after debridement of osteomyelitis and pseudarthrosis remains a major challenge for modern trauma surgery and orthopaedics. Smaller defects up to 4 cm in length are treated with corticocancellous autologous transplants [1, 2], whereas larger defects require major reconstruction, such as the transplantation of vascularised bone [3, 4], the induced membrane concept [5, 6] or bone transport using an Iliazarov fixator [7]. Also, the use of bone replacement materials and allografts has increased in recent years, reducing the need for autologous bone [2, 8].

The induced membrane concept for the reconstruction of long bone defects described by Masquelet et al. is a two-stage surgical technique that successfully repairs bone defects of up to 25 cm [6, 9]. The average defect size treated by the Masquelet technique is 5.5 cm [10]. Traditionally, in the first operation, PMMA (polymethyl methacrylate) cement spacer is inserted into the bone defect, which induces the formation of a membrane needed to avoid ingrowth of soft tissue. After 8–12 weeks, depending on the defect site [10], a second operation is conducted whereby the cement spacer is removed and the induced membrane tube is filled with iliac crest cancellous bone or RIA (Reamer-Irrigator-Aspirator) cancellous bone. Over the course of several months, total bone healing occurs with complete function of the bone.

This concept is now widely used and, with an increase in scientific interest in the induced membrane, has been published in numerous clinical case reports in recent years [11–14] due to its growing importance. However, the membrane or the bone defect healing has only been scientifically studied to a limited extent; therefore, few publications are found in the international literature. Thus far, it has been described that the 1–2 mm thick, sturdy, well-perfused, histologically synovial-like membrane secretes growth factors in rabbit during ectopic induction in the subcutaneous fatty tissue. It also promotes the differentiation or proliferation of osteoblasts and the ability to form bone substance [15]. Histologically, the membrane is composed of fibroblasts, myofibroblasts and collagen, and, as a sign of foreign body reaction, inflammatory cells can be detected, e.g. giant cells. Blood vessels are detectable in all layers of the membrane [13, 16]. When used on bone defects in rats [17] and sheep, bone healing could be achieved with a relatively small number of cases, both with bone replacement materials and cancellous bone [18]. The induced Masquelet membrane supports bone healing in different ways. First, it separates mechanically the bone defect from the surrounding soft tissue and prevents the ingrowth of fibrous tissues into the defect site [9]. Second, the induced Masquelet membrane has pronounced osteogenic and angiogenic properties; it secretes growth factors such as BMP-2, TGF-β1, and VEGF [13] and supports osteoblastic differentiation [19].

Despite the proven beneficial effect of the induced membrane technique on the healing of large bone defects, it would be a tremendous development to shorten the lengthy, two-stage Masquelet procedure. This would save patients the second initial surgical procedure for membrane induction and can possibly be achieved by replacing the induced membrane with a readily available biological membrane.

**Fig. 1:**
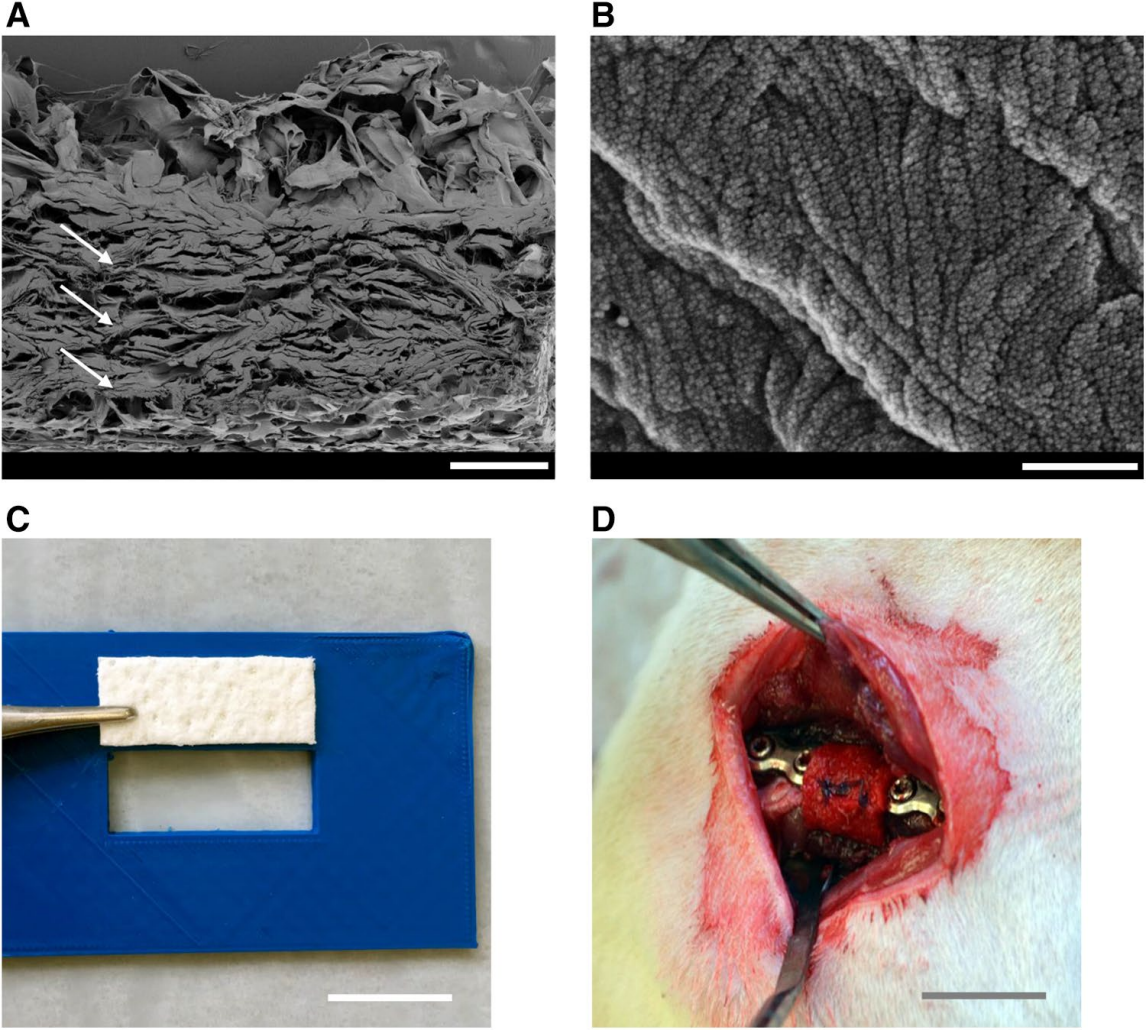
Placement of the acellular dermis into the plate stabilized bone defect, the defect is filled with syngenic spongiosa from donor rats (**a**). SEM-image, cross section of the acellular dermis revealed a highly porous and parallelly organized fibrous macro-structure (arrows, **b**). SEM-image, surface µ-structure of the fibrous macro-structures. Scale bar represents 300 µm in (**a**) and 500 nm in (**b**), 1 cm in (**c**, **d**)

The concept of separation of different tissues compartments to avoid ingrowth of soft tissue in the area of bone defects by the use of artificial membranes is commonly and successfully used in the area of oral surgery, as summarised by Sanz et al. [20] and Tolstunov et al. [21]. The results raise hope that an artificial membrane could also replace the induced membrane for the treatment of long bone defects. This might be beneficial if the replacement membrane offers biochemical and structural characteristics comparable to the induced membrane. Human decellularised dermis is commonly used for the treatment of burn wounds or in repairing abdominal wall defects [22]. It offers similar structural properties compared to the induced membrane with parallelly aligned fibrous layers (Fig. 1), high collagen content [23] and also high tensile strength [24]. Therefore, we aimed to evaluate the effect of a commercially available decellularised human dermis on the healing of a large femoral bone defect compared to the induced membrane technique. A well-established rat model on large femoral defects [25] will be used to compare bone defects treated with the Masquelet technique and bone defects without any additional cover.

## Materials and methods

### Animal care

All animal experiments were performed in accordance with regulations set forth by our institution’s animal care and oversight committee (Project no. FK/1075; Regierungspräsidium, Darmstadt, Germany) in accordance with German law. Fifteen 8–10 week old male Sprague Dawley rats (Janvier Labs, Saint Berthevin, France) weighing approximately 250–320 g were housed in cages with three to four animals per cage. Housing conditions were identical for all rats with temperature (15–21 °C), air flow and light- controlled (14 h day and 10 h night) rooms. Rats were given ad libitum access to rat chow and water. Animal wellbeing was ascertained daily during the first week after surgery and weekly thereafter.

### Human acellular dermis

The human acellular dermis Epiflex^®^ (German Institute for Cell- and Tissue Replacement (DIZG gemeinnützige GmbH, Berlin, Germany) was used to cover the bone defect. Epiflex^®^ is sterilised using a validated, GMP-conformable process and approved as a medicinal product under §21 of the German Medicinal Products Act (license number: 3003749.00.00).

All tissues are acquired from non-profit tissue recovery partners after informed consent. The ADM is produced from skin of serologically screened donors by validated procedures, including decellularisation, sterilisation and preservation of the tissue [26].

### Bone defect model and induction of the Masquelet membrane

The animals were randomly assigned to either control group (no membrane, group 1, *n* = 5), induced membrane group (group 2, *n* = 5) and human decellularised dermis-group (hADM) (group 3, *n* = 5). At baseline, all animals had a comparable age. In group 2, the Masquelet-membrane was induced by inserting a PMMA cement spacer over a period of 3–4 weeks. Animals in groups 1 and 3 were also kept for a corresponding period of time to ensure an identical biological age in all experimental groups.

The femur critical size defect was induced as previously described [27, 28]. In detail, rats were anesthetised by i.p. administration of 2 mL of Ketavet (100 mg/mL) + Rompun (20 mg/mL). The right hind limb of the rat was shaved and aseptically cleaned. A lateral longitudinal incision over the femur was made. The fascia was cut and the muscles were bluntly separated between the musculus quadriceps femoris and the hamstrings. A five-hole locking compression plate (Miniplate ‘Lockingplate LCP Compact Hand 1.5 straight’, Depuy-Synthes, Dubendorf, Switzerland) was placed on the surface of the femoral shaft. Four screws fixed the plate to the bone. Afterwards, the bone cortex was cut by a Gigli saw (RISystems, Davos, Switzerland) and a bone defect of 5–6 mm was created in the midshaft around the middle hole of the plate.

*Group 1, ‘no membrane group’* the defect was filled with freshly isolated and minced syngenic cancellous bone taken from donor rats of the same strain as described [17] and closed afterwards.

*Group 2, ‘induced membrane group’* to induce the Masquelet membrane, the defect was filled with Palacos G (Heraeus Medical, Wehrheim, Germany). In the bone defect, the cement cured with continuous cooling with physiological saline. After this, the wound was closed again [11, 29]. After 3–4 weeks, the femur was re-exposed, the PMMA spacer removed, the fracture ends freshened and the membrane pocket filled with syngeneic cancellous bone and closed [17].

*Group 3, ‘hADM group’* human acellular dermis was cut into pieces of 1.5 m × 0.8 cm using a 3D-printed template and incubated in PBS for 10 min under sterile conditions before being wrapped around the defect site. A pocket was formed and filled with syngenic bone; the dermis was then closed by a suture, thereby enclosing the plate fixateur (Fig. 1).

Finally, the wound over the graft was closed again by assembling the musculi vastus lateralis, biceps femoris and adaption of the wound margins with continuous subcutaneous stitches using a 4/0-monofilament nylon suture. Animals had free access to food and water containing opioids for the first 5 days and were monitored daily for post-operative morbidity. The animals were killed painlessly by an overdose of pentobarbital (500 mg/kg body weight intraperitoneally) after 8 weeks and were weighed afterwards. Femora were then dissected free and all bones were examined macro- and microscopically for signs of infection. Blinded with respect to treatment, all implants were checked for fixation and only in cases where all screws remained firmly tightened was the animal included in further evaluations. Sample processing at 8 weeks: Bones were stored at 4 °C in 70% ethanol until µCT-analysis and subsequent biomechanical testing. After biomechanical testing, bones were wrapped in prewetted gauze and stored at − 80 °C until further processing [27, 28, 30].

### µCT-analysis

To assess bone mineral density and callus volume, µCT analysis was performed with a high-resolution in-vivo-micro-CT Skyscan 1176 (Bruker AXS, Karlsruhe, Germany). The long axis of the femur was lined up orthogonally to the axis of the X-ray beam (Al 0.5 mm; voltage: 50 kV; current: 500 μA; frame average: 7; rotation ra.: 180; rotation st.: 0.5) and the region of interest was placed on the defect. Isotropic voxel size was 18 µm^3^. Two-dimensional CT-images were scanned, reconstructed using a standard back convolution procedure and saved in 3D arrays.

### Biomechanical characterisation

Biomechanical properties of the femur’s defect site were measured by a destructive three-point bending procedure using a material testing machine (Zwickiline Z5.0, Zwick-Roell, Ulm, Germany). The ‘bending until failure’ was performed by lowering a bar onto the femur, using a constant deflection speed of 0.1 mm/s. Load and deflection were recorded continuously. The ultimate load and stiffness (slope of the elastic deformation part of the load/deformation curve) was then calculated using the Testexpert-II software (Zwick-Roell).

### Histological assessment of callus formation

Callus formation and bone maturation were assessed by means of histomorphometric analysis of Movat pentachrome and osteocalcin immunostained decalcified sections taken from the bone defect. For histological evaluation of bone maturation, bones were carefully defrosted and fixed in Zinc-Formal-Fixx, 10% over 20 h (Thermo Electron, Pittsburgh, USA) followed by decalcification for 14 days in 0.25 M Trizma base (Sigma-Aldrich, Taufkirchen, Germany) and 10% EDTA (Sigma-Aldrich), pH-value 7.4. Decalcified bones were paraffin embedded; longitudinal Sections (3 µm) were taken. Movat pentachrome staining of paraffin embedded histological slides was performed as published by Garvey et al. [31] using a staining kit according to the manufacturer’s instructions (Morphisto, Frankfurt, Germany). All slides were analysed using light microscopy. High resolution images depicting the whole defect zone in each case were created by automated stitching of multiple single frames covering the whole defect using the software BZII Analyser (Keyence, Neu-Isenburg, Germany). New bone formation and cartilaginous tissue area were then analysed in the defect site using the software ImageJ (https://imagej.nih.gov/ij/) and the relative tissue positive area of the entire defect zone was calculated.

### Statistics

Results are presented as box plots of the median in diagrams or as mean and standard deviation in the description of the results. A non-parametric Kruskal–Wallis-test with Bonferoni-Holm corrected Conover-Iman posthoc analysis was used for comparisons between the groups using the statistical software Bias 11.10 (Epsilon-Verlag, Darmstadt, Germany). Values are presented either as boxplots of the median in diagrams or, in text, as mean value and standard deviation. *p* values < 0.05 indicate statistical significance. *p* values between 0.05 and 0.1 were rated as statistical trend.

## Results

### Animal care

All animals were included in the evaluation. Screw loosening did not occur and no macroscopically visible side effects of the human acellular dermis were recorded. At the time of kill, the weight of the animals was comparable in all groups. The animals in the no membrane group weighed 618.8 g ± 32.0 g, in the induced membrane group 611.3 g ± 25.3 g and in the dermis group 617.5 g ± 22.2 g.

### Bone healing (descriptive evaluation of µCT analysis)

**Fig. 2:**
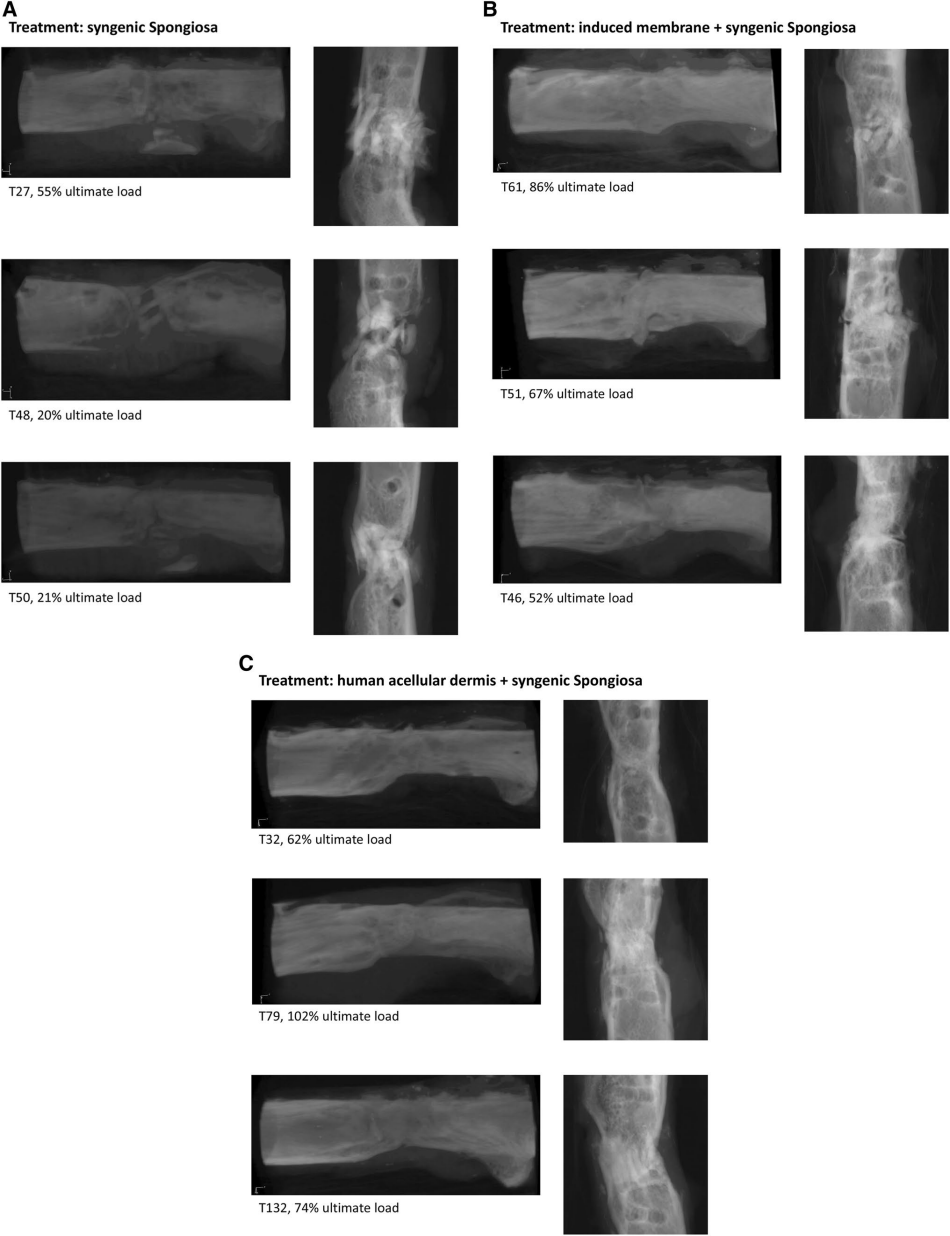
Three-dimensional reconstruction of representative serial µCT images (left column) and 2D slices of the scans (right column) of femoral defects treated with either syngenic bone (**a**), combination of induced membrane and syngenic bone (**b**) and combination of human acellular dermis and syngenic bone (**c**)

Bone was radiologically healed in 5 of 5 animals in the dermis group, 4 of 5 animals in the induced membrane group, and 0 of 5 animals in the no membrane group. In the group without membrane, we were able to see callus formation in all µCT scans, but no complete ossification. The induced membrane group showed ossification in more cases. However, the best ossification in all cases, modelling and start of remodelling, was observed in the dermis group (Fig. 2).

**Fig. 3:**
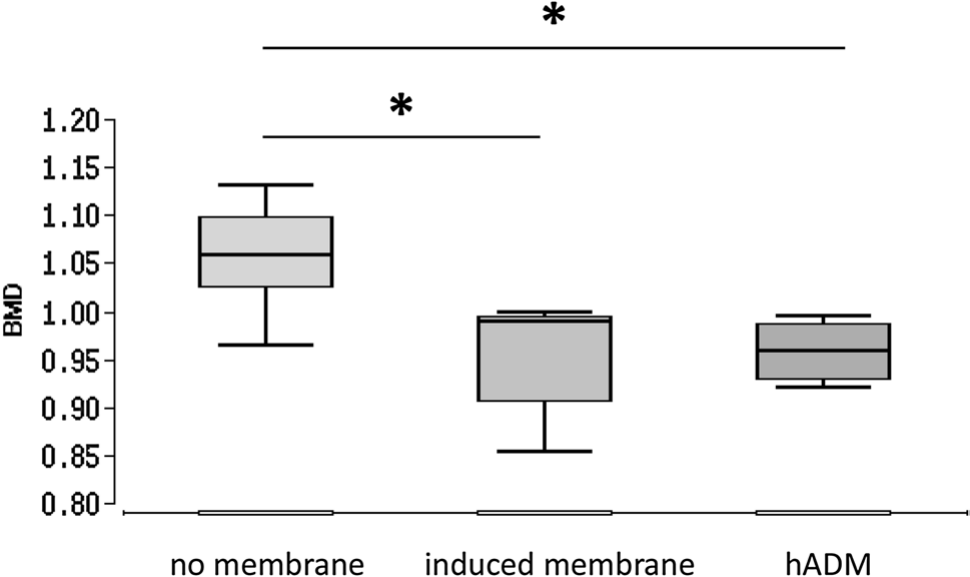
Assessment of bone mineral density using μCT technique [relative, dimensionless units]. Significant differences between no membrane vs. induced membrane and no membrane vs. hADM. No significant differences between induced membrane vs. hADM. *p < 0.05

Bone mineral density (BMD) was assessed using µCT technique in relative, dimensionless units. The significantly highest BMD was found in the no membrane group 1.1 ± 0.05 (*p* < 0.05 vs. induced membrane and vs. hADM group). No significant differences were observed between the induced membrane (1.0 ± 0.06) and the dermis group (0.9 ± 0.03) (Fig. 3).

### Biomechanics

**Fig. 4:**
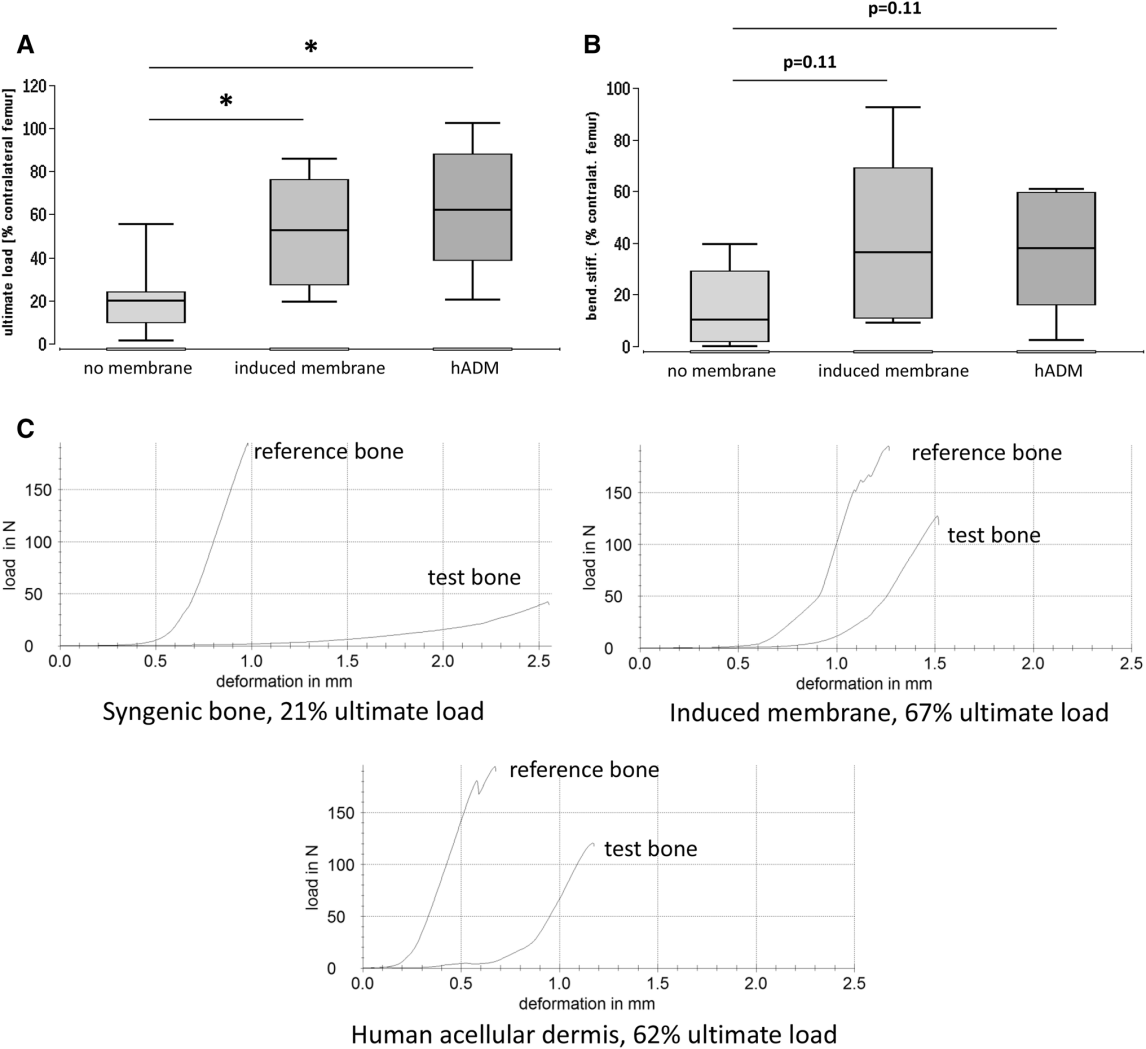
Ultimate load (**a**) and bending stiffness (**b**) of the defect zone treated with syngenic spongiosa, induced membrane filled with syngenic spongiosa (Masquelet technique) or human acellular dermis (hADM) filled with syngenic spongiosa. Biomechanical properties of the defect zone were measured by means of three-point bending test eight weeks after transplantation. Representative force/deformation curves of femora obtained from animals that received either solely syngenic spongiosa, induced membrane and syngenic spongiosa or human acellular dermis and spongiosa are presented in (**c**). The callus was not bridged in the control group. The force/deformation curve of the respective healthy contralateral femur is shown for comparison in each example. Peak of the curves indicate the ultimate load of the bone samples. *p < 0.05

A healthy bone taken from the contralateral side resisted forces of up to 161.4 N ± 24.5 N [11, 32]. Bone ultimate load was measured in % for each measured contralateral femur with the highest resistance in hADM group (63.2% ± 29.6%, *p* < 0.05 vs. no membrane). Similar results were found in the induced membrane group (52.1% ± 25.8%, *p* < 0.05 vs. no membrane), while the no membrane group presented significantly decreased resistance (21.5% ± 16.8%) (Fig. 4).

Figure 4 displays the bending stiffness in % for each contralateral femur. We calculated *p* values and local p* values without α-correction. Similar results were observed in the hADM group (37.9% ± 23.8%, *p* = 0.11, p* = 0.037 vs. no membrane) and the induced membrane group (39.5% ± 33.4%, *p* = 0.11, p* = 0.048 vs. no membrane). The lowest results were shown in the no membrane group (14.3% ± 14.5%) (Fig. 4).

The representative force/deformation curves of femora obtained from animals that received either solely syngenic spongiosa, induced membrane and syngenic spongiosa or human acellular dermis and spongiosa are presented in Fig. 4. The force/deformation curve of the respective healthy contralateral femur is shown for comparison in each condition. The peak of the curves indicates the ultimate load of the bone samples.

### New bone formation

**Fig. 5:**
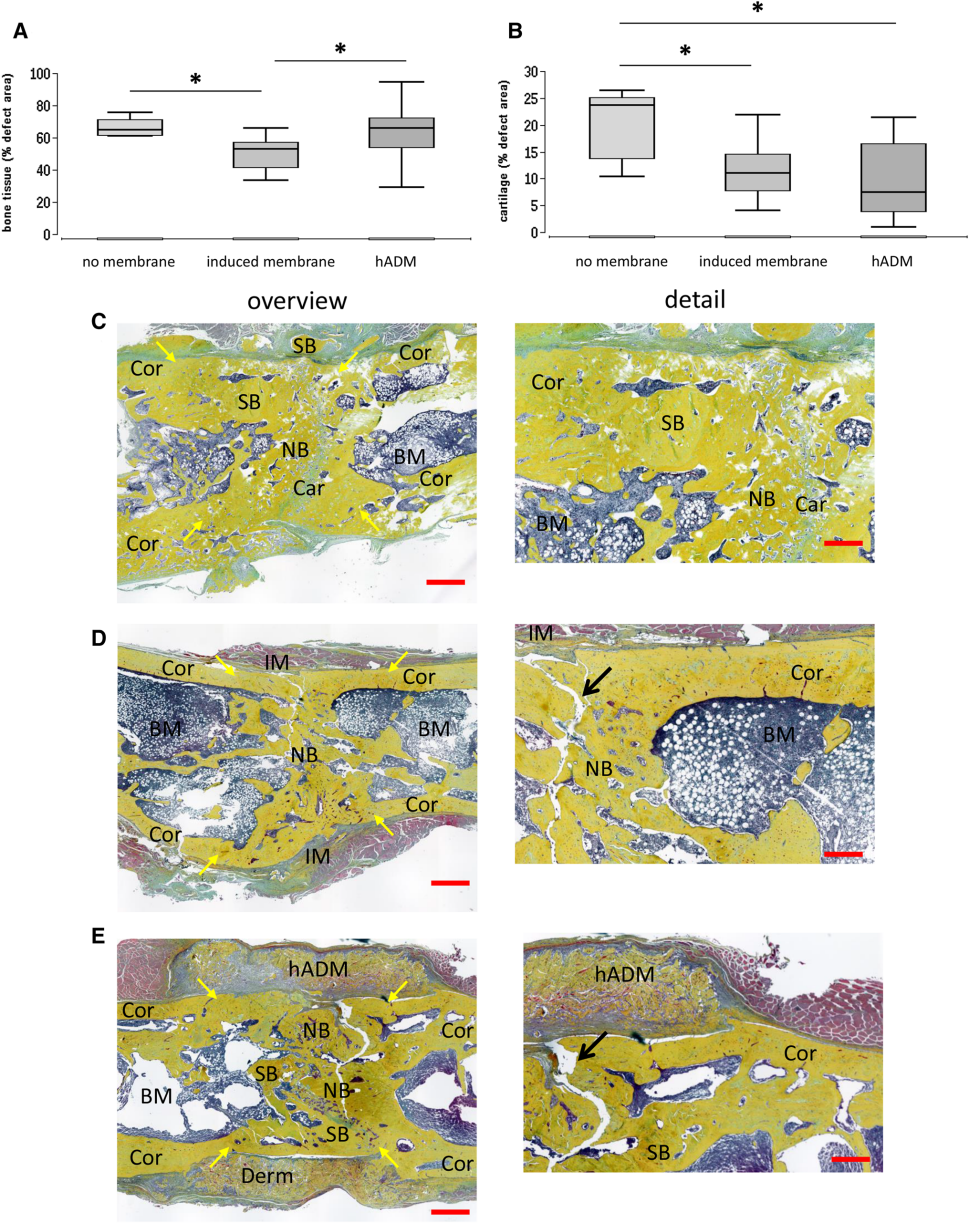
Percentage of bone tissue (**a**) and cartilage (**b**) in the defect area as assessed by histomorphometric analysis of Movat’s pentachrome stained histological slices eight weeks after surgery. Representative images of Movat’s pentachrome staining of defects transplanted solely with syngenic bone (**c**), with induced membrane and syngenic bone (**d**) and human acellular dermis and syngenic bone (**e**) are shown. Left column provides an overview of the whole defect, whereas corresponding detailed enlargements were presented in the right column. Bone tissue appears yellow, cartilaginous tissue appears cyan. Bone was used for biomechanical testing before histological preparation. Thus, fractured areas (indicated by arrows) are seen in samples presented in this figure. *BM* bone marrow, *Car *cartilaginous tissue, *Cor *corticalis, *hADM *human acellular dermis, *IM *induced membrane, *NB *newly formed bone tissue, *SB *transplanted syngenic bone. Yellow arrows indicate edges of the original defect. Red bars represent 1 mm (left column), respectively, 250 µm (right column). *p < 0.05

The percentage of bone tissue and cartilage in the defect area was assessed by histomorphometric analysis of Movat’s pentachrome stained histological slices at 8 weeks after surgery. Newly formed bone had replaced 66.2% ± 5.8% of the bone defect in the no membrane group, compared to 50.7% ± 10.0% (*p* < 0.05) in the induced membrane group. In the hADM group, newly formed bone covered 64.2% ± 17.2% of the defect area (*p* < 0.05 vs. induced membrane) (Fig. 5).

The mean cartilage formation was 20.3% ± 6.5% of the defect area in the no membrane group, which is significantly more compared to the induced membrane (11.8% ± 5.1%, *p* < 0.05) and hADM group (9.1% ± 6.9%, *p* < 0.05) (Fig. 5).

## Discussion

The therapeutic effect of a human acellular dermis on the healing of a large (5 mm) rat femoral bone defect transplanted with syngeneic spongiosa was investigated. This was compared to the two-stage Masquelet-induced membrane technique and an equally treated, but uncovered, bone defect. A comparably good and partially improved bone healing was achieved in the one-stage technique using the human acellular dermis compared to the two-stage Masquelet technique. Both groups showed significantly improved healing results in comparison to the uncovered bone defects 8 weeks after surgery.

A review of the current literature shows that the Masquelet technique is effective for the treatment of large bone defects. For example, Raven et al. were able to achieve good healing results in patients with atrophic and/or infected pseudarthrosis [33]. The use of this technique also provided acceptable levels of healing in the military setting for patients with open tibial fractures; in the latter case, prior elimination of infections was a major challenge [34].

In addition to clinical analyses, research is increasingly being published to improve the induced membrane technique. Two strategies can be identified: the first is the membrane properties using PMMA additives such as antibiotics [11], while the second is the use of other spacer materials, such as titanium, where surfaces can be additionally modified by microstructures. In the latter study, it was also possible to demonstrate that these spacer-induced modifications of the membrane have a significant influence on the healing result in the rat femoral bone defect (6 mm) [35].

On the other hand, it is possible to directly support the formation of the induced membrane by auxiliary structures. Liu et al. combined an artificial dermis with the PMMA spacer using a rabbit femoral defect model to aid in the formation of the induced membrane. Histological analysis showed that the dermis had completely disappeared 4 weeks after the operation, but, in this experimental group, the vascularisation of the induced membrane was significantly increased [36].

However, the two-stage Masquelet concept remains untouched. In our present work, this was avoided using a substitute material instead of the induced membrane. Criteria for the selection of the replacement material were structural and biochemical homologies to the induced membrane. To avoid graft versus host reactions, a cell-free preparation should be sought. A human acellular dermis (‘Epiflex’), which is used as a clinically approved product in the treatment of large (burn) wounds, comes close to the desired criteria. However, unlike the induced membrane at the time of the second operation (removal of the spacer, filling with bone material), cell colonisation, vascularisation and growth factors, such as, for example, BMP-2 and TGF-β [13, 29], factors believed to support bone healing, were not present in the acellular dermal graft. In pre-tests we were able to see that the material used to absorb significant blood volume in the hydrated state, hence, during implantation, a certain biologisation of the membrane takes place. Other researchers have also shown that the acellular dermal matrix can be quickly vascularised [37], but the relevance of vascularisation of the induced membrane for bone healing is not clearly established. Although vascularisation of the induced membrane has been demonstrated in previous studies [11, 17, 29], no significant vascular connection between the induced membrane and the defect region could be histologically detected [17]. This suggests that this mechanism is unlikely to be relevant to supporting bone defect repair through the membrane. Whether or not the acellular dermis is vascularly connected to the defect zone should the subject of future investigations. These considerations suggest that the barrier function of the induced membrane or acellular dermis may play an important role in the bone healing process. It can be assumed that the ingrowth of fibrous tissue from the surrounding soft tissues into the defect and, thus, the formation of pseudarthrosis is effectively prevented by the membrane. This assumption is supported by extensive experience in oral surgery [21, 38]. Furthermore, in the present study, it could be demonstrated histologically and radiologically that both examined defect covers kept the transplanted syngeneic cancellous bone local. In the group without defect coverage, cancellous bone in close proximity, but outside the defect zone, as well as displacements of the transplanted cancellous bone from the defect area, could be observed. As a result, the transplants lost contact with each other and with the ends of the fractures, which may have led to the observed less stable or incomplete bone healing.

In contrast to this, histologically the bone mass in uncovered defects was significantly increased compared to defects with induced membrane. This surprising finding could be explained by advanced bone healing in the induced-membrane group. Both histologically and radiologically, clear signs of progressive bone reshaping were evident in this group (and, in part, also in the acellular dermis group). The bone mass in the defect area was reduced, a medullary cavity was emerging and the cortical structures were reinforced (Fig. 5). Delayed bone healing in the group without defect envelopment is substantiated by further histological evaluations. Thus, the area of osteochondral bone regeneration was significantly higher in this group than in the two comparison groups, in which this stage of new bone formation was presumably already subsiding. The increased bone mineral density in the group without defect envelopment was likely due to the transplanted bone material and not indicative of newly formed mineralised bone tissue. This assumption is supported by the radiological images in which the transplanted bone material was visible due to its high radiopacity. This also indicates a lack of integration and remodelling of the transplanted material in the newly formed bone.

## Limitations

The present study is descriptive and no mechanistic aspects have been examined. Presently, this technique is likely to be limited to aseptic bone defects only. A preceding antibiotic antimicrobial therapy of the infectious source would ultimately correspond to the effort of the classic two-time Masquelet technique. Additional information regarding efficacy may be gathered in a large animal model.

## Conclusion

Assuming that the presented one-stage membrane concept can be transferred into surgical practice, the procedure presented here is of high clinical relevance in the field of trauma surgery and orthopaedics. By eliminating the first operation to induce the membrane, the patient’s suffering is reduced, the treatment time is significantly shortened and, ultimately, the cost of treatment is reduced.
